# Inequalities and mental health during the Coronavirus pandemic in the UK: a mixed-methods exploration

**DOI:** 10.1186/s12889-023-16523-9

**Published:** 2023-09-20

**Authors:** Chiara Lombardo, Lijia Guo, Susan Solomon, David Crepaz-Keay, Shari McDaid, Lucy Thorpe, Steven Martin, Ann John, Alec Morton, Gavin Davidson, Antonis A. Kousoulis, Tine Van Bortel

**Affiliations:** 1 Cambridge Public Health Interdisciplinary Research Centre, Department of Psychiatry, University of Cambridge School of Clinical Medicine, Cambridge Biomedical Campus, Box 113, Cambridge, CB2 0SR UK; 2 Mental Health Foundation, Studio 2, 197 Long Lane, London, SE1 4PD UK; 3 Leicester School of Allied Health Sciences, Faculty of Health and Life Sciences, De Montfort University, Gateway House, Leicester, LE1 9BH UK; 4 Health Data Research UK, Swansea University Medical School, Singleton Park, Swansea, SA2 8PP UK; 5 Department of Management Science, Strathclyde Business School, University of Strathclyde, 199 Cathedral Street, Glasgow, G4 0QU UK; 6 School of Social Sciences, Education and Social Work, Queen’s University Belfast, Belfast, BT7 1NN UK

**Keywords:** Coronavirus, Covid-19, Pandemic, Inequalities, Inequity, Social determinants, Mental health, Adult population, United Kingdom

## Abstract

**Background:**

The World Health Organisation declared the novel Coronavirus disease (COVID-19) a global pandemic on 11th March 2020. Since then, the world has been firmly in its grip. At the time of writing, there were more than 767,972,961 million confirmed cases and over 6,950,655 million deaths. While the main policy focus has been on controlling the virus and ensuring vaccine roll-out and uptake, the population mental health impacts of the pandemic are expected to be long-term, with certain population groups affected more than others.

**Methods:**

The overall objectives of our ‘Coronavirus: Mental Health and the Pandemic’ study were to explore UK adults’ experiences of the Coronavirus pandemic and to gain insights into the mental health impacts, population-level changes over time, current and future mental health needs, and how these can best be addressed. The wider mixed-methods study consisted of repeated cross-sectional surveys and embedded qualitative sub-studies including in-depth interviews and focus group discussions with the wider UK adult population. For this particular inequalities and mental health sub-study, we used mixed methods data from our cross-sectional surveys and we carried out three Focus Group Discussions with a maximum variation sample from across the UK adult population. The discussions covered the broader topic of 'Inequalities and mental health during the Coronavirus pandemic in the UK’ and took place online between April and August 2020. Focus Groups transcripts were analysed using thematic analysis in NVIVO. Cross-sectional survey data were analysed using STATA for descriptive statistics.

**Results:**

Three broad main themes emerged, each supporting a number of sub-themes: (1) Impacts of the pandemic; (2) Moving forward: needs and recommendations; (3) Coping mechanisms and resilience. Findings showed that participants described their experiences of the pandemic in relation to its impact on themselves and on different groups of people. Their experiences illustrated how the pandemic and subsequent measures had exacerbated existing inequalities and created new ones, and triggered various emotional responses. Participants also described their coping strategies and what worked and did not work for them, as well as support needs and recommendations for moving forward through, and out of, the pandemic; all of which are valuable learnings to be considered in policy making for improving mental health and for ensuring future preparedness.

**Conclusions:**

The pandemic is taking a long-term toll on the nations’ mental health which will continue to have impacts for years to come. It is therefore crucial to learn the vital lessons learned from this pandemic. Specific as well as whole-government policies need to respond to this, address inequalities and the different needs across the life-course and across society, and take a holistic approach to mental health improvement across the UK.

**Supplementary Information:**

The online version contains supplementary material available at 10.1186/s12889-023-16523-9.

## Introduction

On 11^th^ March 2020, the World Health Organisation declared a global pandemic of the novel Coronavirus (SARS-Cov-2) and COVID-19 disease. At the time of writing, this pandemic accounts for more than 767,972,961 million confirmed cases and over 6,950,655 confirmed deaths worldwide [1]. Whilst the physical health impacts are visibly enormous, the gravity of the pandemic’s mental health implications are, thus far, less tangible and require further investigation and action [2]. Meantime, both direct and indirect mental health consequences of the pandemic are being observed across the UK population including reports of increased depression, anxiety [3–5], poor sleep and loneliness [6–8], and a higher frequency of abuse and self-harm among certain groups [4, 9].

The repeated cross-sectional survey data from our mixed-methods ‘Coronavirus: Mental Health in the Pandemic’ study [10] found that key indicators of distress among UK adults worsened during the first nine months of the pandemic, with the proportion of people reporting feelings of loneliness and being unable to cope with stress both increasing from 10% in March 2020 to 25% in November 2020 [9, 11, 12].

Whilst the global vaccine roll-out is firmly underway to protect us against the worst effects of COVID-19, there is no vaccine to safeguard us from the mental health consequences of the pandemic. Instead, we need to focus on prevention and early intervention – including addressing the underlying mental ill-health causes, such as unemployment, bad employment, poor housing, poverty, and social isolation. This is now even more important as the pandemic has not only exacerbated existing socio-economic disadvantages, healthcare inequalities and traumas experienced by people with mental health problems, it has also created new ones [12–16].

Further, there is increasing evidence that certain sections of the population are differently affected by the Coronavirus pandemic, depending on their circumstances. The pandemic seems to have widened mental health inequalities too: groups that had the poorest mental health pre-pandemic also experienced the largest mental health deterioration during lockdown [12, 17–19]. The economic effects are also variable. There are signs of increasing economic inequality, with more people with lower personal incomes reporting reduced household income because of Coronavirus measures such as lockdowns, fewer working hours, and being less able to save for the future, while fewer people with higher incomes have been impacted financially [20, 21]. Estimates indicate that more than half a million people in the UK are likely to experience mental health problems as a direct result of the economic impact of the pandemic [22].

### Study aims

Our mixed-methods ‘Coronavirus: Mental Health in the Pandemic’ study aims to gain insights into the mental health impacts, dynamics, and experiences of the Coronavirus pandemic and associated measures on the UK adult population, how these change over time, what the current and future mental health needs are, and how best to address these within context [3].

The specific objectives of this mix-methods sub-study were to describe people’s concerns, emotions and coping strategies through our quantitative cross-sectional survey data. And, subsequently, utilising our qualitative Focus Group data, gaining a deeper understanding of these experiences and perceptions, especially regarding emerging and widening inequalities during the Coronavirus pandemic in the UK and their impacts on mental health, how people coped with this, and how this can best be addressed in the shorter and longer-term.

### Research questions

- What are UK adults’ experiences and perceptions of existing and emerging inequalities and their impacts on mental health during the Coronavirus pandemic and associated measures?
- What is the nature of these inequalities?
- What are the reasons for these inequalities?
- How do people cope with the challenges of the pandemic? Which coping mechanisms and measures worked well and which did not work well?
- What can be done to address these inequalities and to support people’s mental health in the shorter and longer-term?

## Methods

A mixed-methods design was chosen for this sub-study to enable an in-depth exploration of the emerging inequalities and their impact on mental health.

This present research forms part of the wider UK ‘Coronavirus: Mental Health in the Pandemic’ study, where regular UK-wide representative cross-sectional online population surveys were collected. Non-probability quota sampling was used and was repeated across 13 waves starting from March 2020 through to September 2021. Each wave aimed to select a national sample representative of the adult (18 >) population living in the UK, designed to be representative of the population based on age, sex, education and social class. Participants were different in each wave but taken from the same panel and representative of the UK adult population. The tailored online survey questionnaire (please refer to the Supplementary material 1 for the copy of the survey questionnaire) was administered by YouGov to 2 400 000 + individuals drawn from across the entire UK who agreed to take part in research surveys. Repeated cross-sectional surveys are an ideal method to provide good estimates for the current population (at each cross-sectional survey) and the changes over time (across the repeated cross-sectional surveys) at population level.

Qualitative research (individual and group interviews) was tailored around our emerging survey findings, combined with relevant emerging literature and media reporting in relation to the mental health impacts of the Coronavirus pandemic in the UK. Further details can be found in our published study protocol [10].

Data for the ‘Coronavirus: Mental Health in the Pandemic’ study were first collected shortly before the first UK-wide lockdown was announced and repeated approximately every 4-6 weeks, and/or at crucial points in time.

The mixed methods sub-study here presented comprises of:

- Three survey data waves with wave 2, wave 4, and wave 6 (conducted in April 2020, June 2020 and August 2020), corresponding with the timing of the three respective Focus Groups. Participants signed up to YouGov to participate in surveys and they read and agreed to the terms and conditions of use and privacy policy before responding [23]. The survey was designed to gage the extent and nature of the mental health experiences and dynamics of the coronavirus pandemic and coping strategies as well as changes over time. Results from the survey waves informed the topics and structure of the subsequent Focus Groups.
- Three Focus Group Discussions (FGD) around the topic ‘Inequalities and mental health during the Coronavirus pandemic in the UK’, with an additional focus in each FGD on specific key aspects within this wider topic, being: (1) Socio-economic inequalities and mental health experiences, (2) Divergence of inequality experiences, (3) Mental health resilience and coping strategies.

The FGDs were designed to better understand and further contextualise emerging quantitative survey findings. For this mixed-methods sub-study, they were specifically designed to explore the experiences of and perceptions around inequalities that were not otherwise addressed in, or explained through, the quantitative survey data. The FGDs helped to provide a rapid policy response, and explore specific relevant issues. Additional qualitative research was carried out in the context of ethnic minorities, older people and young people [23–26].

Our FGDs enabled us to explore in-depth and in an organised manner, the experiences and perspectives of the UK adult population regarding various aspects of the mental health impacts of the Coronavirus pandemic and associated measures.

### Focus group discussions

Details of each FGD topic and timing can be found in Table 1. The FGDs were carried out entirely virtually via online facilities (ZOOM or Microsoft Teams), as various restrictions were in place throughout this period starting with the first UK national lockdown from 23^rd^ of March 2020 to 4^th^ of July 2020^1^ Each FGD lasted for approximately one hour and 30 min and were all co-facilitated by two experienced Co-Chairs. Meetings were video-recorded (upon consent of all participants) and notes were also taken by hand by a silent observer (TVB) which was made clear to the participants. The FGDs were transcribed using an authorised transcription service. The FGD facilitator (CL) and silent observer (TVB) were qualitative experts in the field of mental health, and trained in safeguarding procedures in research and data protection for field notes and data collection.

**Table 1:** Details of the three focus group discussions

Focus group discussion (FGD)	Sub-topic	Context	Timing
1 Inequalities and mental health	Socio-economic inequalities and mental health experiences	Coronavirus pandemic and related measures in the UK at that time	April 2020 (during the first UK-wide lockdown)
2 Inequalities and mental health	Divergence of inequalities and mental health experiences		June 2020 (upon gradual lifting of first UK lockdown)
3 Inequalities and mental health	Mental health resilience and coping strategies	Lifting of restrictions	August 2020 (upon first UK lockdown fully lifted)

### Recruitment

We used a purposefully selected ‘maximum variation sample’ of people drawn from the UK adult population with lived mental ill-health experiences, advocates for those with mental ill-health problems, and experts in the mental health field, in order to capture as wide a variety of views, perceptions and experiences as possible [27].

Following the repeated cross-sectional survey findings, we held three virtual FGDs on topics of importance and concern in relation to ‘Inequalities and mental health during the Coronavirus pandemic in the UK’.

Potential participants were approached through gatekeeper organisations such as third sector organisations, using a pool of existing contacts for initial approach. Potential participants received an invitation email with the study background information and the topic for the FGD. When potential participants wished for more information and/or to participate in the FGD, they were contacted by a designated study researcher. Participants subsequently received further information about the FGD and – upon agreeing to participate – a Consent Form to provide written understood consent prior to any virtual meetings. Participants were given at least 24 h to decide whether or not they wanted to take part. Participants were also reassured about the confidential nature of the study and that they could withdraw consent at any time. They received reimbursement for the time to participate in the study.

### Focus group discussion structure

Whilst the overarching topic for each FGD was around ‘Inequalities and mental health during the Coronavirus pandemic in the UK’, each FGD also had a specific sub-focus (as demonstrated in Table 1).

Each FGD followed a similar format and structure. FGD 1 and FGD 2 started with a brief presentation of survey data by one of the chairs, followed by a discussion organised around a topic guide with semi-structured open-ended questions around the overall topic and selected sub-topics. Following participants’ feedback, FGD 3 had a slightly different format, with the presentation of our survey results at the end.

The topics for each FGD were tailored around areas of interest and/or concern emerging from each ‘wave’ of cross-sectional survey data which were discussed during weekly research team meetings attended by all the authors, as reflected in Table 1 above. Topic guides reflected the results of each wave and the team meetings discussions as described above. We adapted the topic guide to make the FGDs more relevant and complement the findings of the quantitative survey waves. The topic guides can be found in Supplementary material 2.

### Focus group participants’ characteristics

All participants were aged 18 or over and came from across the UK. FGD 1 was attended by 14 participants (9 women and 5 men), FGD 2 counted 6 participants (3 women and 3 men), and FGD 3 was attended by 12 participants (6 women and 6 men). As each FGD had a specific (different) sub-focus, some of the participants volunteered to attend more than one FGD (as set out in Table 3). The participants represented people with mental ill-health experiences or belong to specific population groups such as those affected by self-injury, older people, rural mental health awareness campaigners, bipolar organisation, inequality groups such as LGTB + and minority backgrounds, survivors of domestic violence groups, parents. Those who had experienced mental health problems did not disclose the nature of their experience, apart from one person who declared to be a survivor of domestic abuse. Those who were professionals, were in a senior/managerial position in either local or national mental health organizations. Table 2 below presents a distribution of the participants for each FGD.

**Table 2:** Demographic characteristics of FGDs participants

Primary sample criteria	Sample characteristics	Achieved sample
**FG1**		
** Gender**	Male	5
	Female	9
** Country**	England	8
	Northern Ireland	1
	Scotland	1
	Wales	2
** Background**	Professional	7
	Carer	1
	Lived experience	2
	L/P	3
	L/P/C	1
**Tot 14**		
**FG2**		
** Gender**	Male	3
	Female	3
** Country**	England	4
	Northern Ireland	0
	Scotland	1
	Wales	1
** Background**	Professional	2
	Carer	0
	Lived Experience	0
	L/P	4
	L/P/C	0
**Tot 6**		
**FG3**		
** Gender**	Male	6
	Female	6
** Country**	England	8
	Northern Ireland	2
	Scotland	0
	Wales	2
** Background**	Professional	5
	Carer	1
	Lived experience	2
	L/P	5
	L/P/C	1
**Tot 12**		

*Legend*: *L/P* Lived experience and Professional, *L/P/C* Lived experience, Professional and Carer

We invited each participant to attend every FGD each time, however we received declines due to a number of reasons** (**such as people falling ill, people needing to drop out of the study due to Long COVID infection, caring responsibilities or for other reasons, such as specific interest to the topic). Some participants were able to attend all FGDs, some only two and some attended only one. A full summary of attendance is presented in Table 3, together with details of their background.

**Table 3:** Participants attendance to FGDs

**Participant characteristics **				**FGD attended**
*Participant number*	*Gender*	*Country *	* Background *	
P01	Female	England	Director of a local mental health charity branch	1
P02	Female	England	Lived experience; Artist	1, 2, 3
P03	Female	England	Works for a mental health charity	1
P04	Female	England	Works for national charity supporting people who survived suicide	1
P05	Female	England	Lived experience; Carer; Independent mental health adviser	1, 2
P06	Female	England	Worked in mental health and psychiatric services	3
P07	Female	England	Peer support coordinator	3
P08	Male	England	Lived experience; NHS Mental Health Equalities Taskforce	1, 2, 3
P09	Male	England	Runs an over-60s membership group	1, 2
P10	Male	England	Mental Health Promotion Trainer	1, 3
P11	Male	England	Lived experience	3
P12	Male	England	Lived experience; CO in a mental health charity	3
P13	Male	England	Peer support volunteer	3
P14	Male	Scotland	Chair of a mental health charity	1, 2, 3
P15	Female	Wales	Domestic violence survivor; professional photographer	1, 2
P16	Female	Wales	Lived experience	1
P17	Male	Wales	Carer of someone with mental illness	1
P18	Male	Wales	Lived experience; Volunteer in a mental health charity	1
P19	Female	England	Research occupational therapist working in mental health services	3
P20	Male	Wales	Lived experience; Volunteer in a mental health charity; Runs a theatre company	3
P21	Male	Northern Ireland	Lived experience; Peer researcher	3
P22	Female	Northern Ireland	Mental Health charity manager; Blogger	1

### Qualitative data analysis

The data underwent Thematic Analysis following Braun and Clarke [28]. Transcripts were read and re-read and line-by-line coding was carried out using NVivo 12 software (CL). Each code’s data was checked for consistency of interpretation and discussed with qualitative research team members (TVB, SS) and re-coded as necessary. The semi-structured topic guide research questions were used as an overall framework for the higher-order themes, together with the interview notes and a preliminary scan of the transcripts. Further confirmation of themes took place through team discussions with qualitative leads CL and TVB. The researchers who conducted the analysis were experienced in the field of public mental health and qualitative research, with different degrees of expertise in the field of health inequalities.

### Ethics approvals

Ethics approvals were obtained from the University of Cambridge Psychology Research Ethics Committee (No. PRE 2020.050) and from De Montfort University Faculty of Health and Life Sciences Research Ethics Committee (No. REF 422991).

### Quantitative survey findings that informed FGDs

#### Data analysis

Three waves of survey data with wave 2, wave 4, and wave 6 (conducted in April 2020, June 2020 and August 2020), corresponding with the key timings of the first UK-wide lockdown period (from the beginning of lockdown until full lifting of restrictions). The FGD topic guides were informed by the survey findings relevant to inequalities and divergences of experiences during the pandemic. We presented the descriptive statistics of the overall situation regarding socio-economic inequalities, the divergence of emotional experiences, and coping for the three key timings respectively. Our survey findings subsequently informed our three FGDs topic guides. In what follows, the results from wave 2, 4, and 6 respectively are presented.

## Results

### Survey results

#### Wave 2*:* Socio-economic inequalities and mental health

We analysed the socio-economic inequalities during the pandemic using the survey data collected at wave 2 (corresponding to the time of Focus Group 1). We conducted our ‘wave 2’ survey data collection in April 2020 (during the first UK-wide lockdown) with a total of 2221 participants. (Table 4 below shows the demographic characteristics of the participants).

**Table 4:** Descriptive statistics of participants at wave 2

Participant characteristics	Category	N	Percent
Gender	Female	1,204	54.21
	Male	1,017	45.79
Age group	18–24	215	9.68
	25–34	355	15.98
	35–44	368	16.57
	45–54	350	15.76
	55 +	933	42.01
Social grade	C2DE, working	903	40.66
	ABC1, middle	1,318	59.34
Work status	Working full time	881	39.67
	Working part time	365	16.43
	Full time student	123	5.54
	Retired	564	25.39
	Unemployed	77	3.47
Marital status	Not working/Other	211	9.5
	Married/ Civil Partnership	1,036	46.65
	Living as married	298	13.42
	Separated/ Divorced	208	9.37
	Widowed	80	3.6
	Never Married	584	26.29
Country	England	1,876	84.47
	Wales	109	4.91
	Scotland	172	7.74
	Northern Ireland	64	2.88

**Fig. 1:**
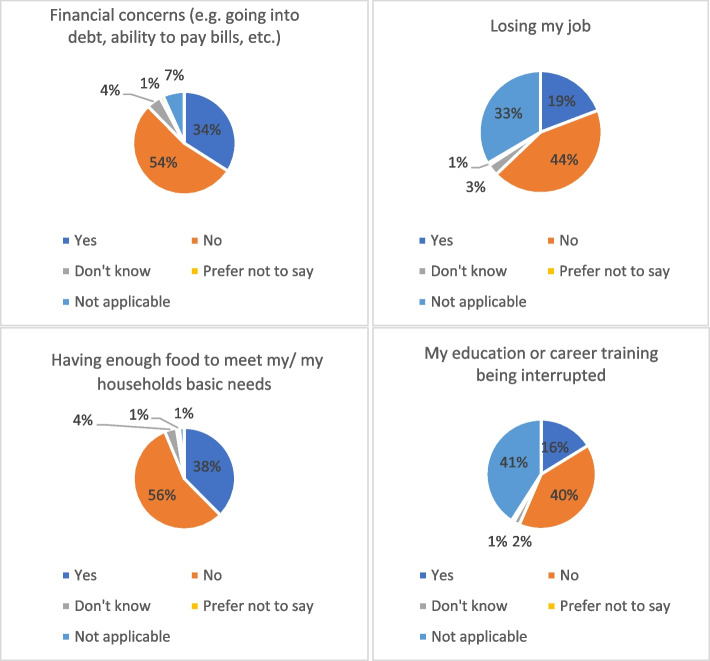
Financial concerns and socio-economic inequalities (overall situation)

In the survey, we asked participants the question: “Have you been worried about any of the following as a result of the Coronavirus (COVID-19) pandemic in the past 2 weeks?” As demonstrated in Fig. 1, among a total of 2221 participants, people’s main worries revolved around financial concerns (34%), losing job (19%), having enough food to meet my/my household’s basic needs (38%), and worries about education or career training being interrupted (16%).

Following the results of survey wave 2, we conducted our first FGD regarding “Socio-economic inequalities and mental health” (reported further in this paper). We based the FGD topic guide on the key survey findings, which indicated that the impact of financial and socio-economic inequalities on mental health during the pandemic became evident.

#### Wave 4: Diverging inequalities and mental health experiences

We then analysed the diverging experiences during the pandemic using the survey data collected at wave 4 (corresponding to the time of Focus Group 2). We conducted our ‘wave 4’ survey data collection in June 2020 (upon gradual lifting of the first UK lockdown) with a total of 4382 participants. (The Table 5 below shows the demographic characteristics of the participants).

**Table 5:** Descriptive statistics of participants at wave 4

Participant characteristics	Category	N	Percent
Gender	Female	2,382	54.36
	Male	2,000	45.64
Age group	18–24	338	7.71
	25–34	671	15.31
	35–44	742	16.93
	45–54	780	17.8
	55 +	1,851	42.24
Social grade	C2DE, working class	1,707	38.95
	ABC1, middle class	2,675	61.05
Work status	Working full time	1,758	40.12
	Working part time	585	13.35
	Full time student	159	3.63
	Retired	1,215	27.73
	Unemployed	159	3.63
	Not working/Other	506	11.55
Marital status	Married/ Civil Partnership	2,123	48.45
	Living as married	585	13.35
	Separated/ Divorced	390	8.9
	Widowed	153	3.49
	Never Married	1,101	25.13
Country	England	3,666	83.66
	Wales	220	5.02
	Scotland	378	8.63
	Northern Ireland	118	2.69

**Fig. 2:**
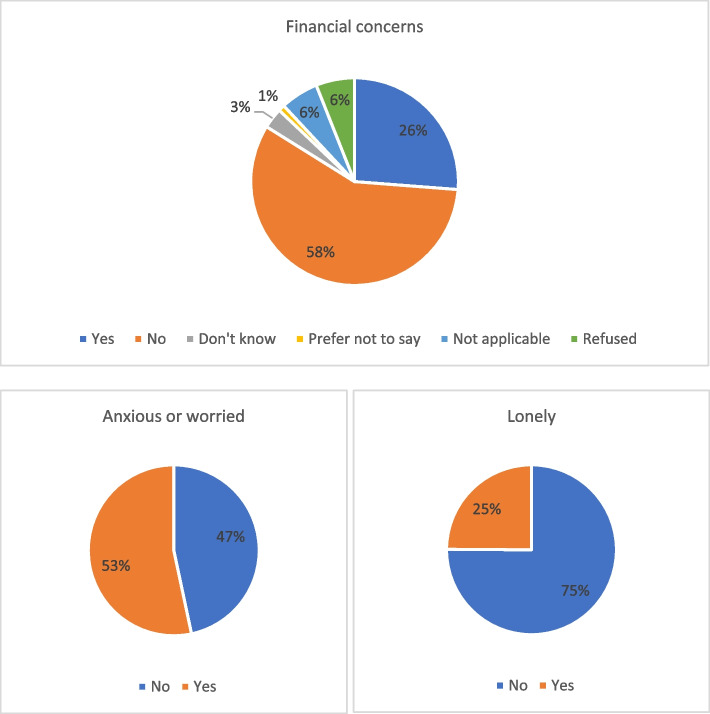
Financial concerns and diverging emotional experiences (overall situation)

At survey data collection wave 4, we asked the participants about their worries and concerns. As demonstrated in the Fig. 2, among a total of 4382 participants, around 26% of them had financial concerns. We also asked participants about their emotional experiences. Among a total of 4382 participants, around 53% of them indicated anxiety or worries, and 25% experienced loneliness.

Following the wave 4 survey results, we conducted our second FGD regarding “Diverging inequalities and mental health experiences” (reported further in this paper). We based the topic guide on the findings from the survey data, which pointed out people’s financial concerns and divergence of emotional experiences during the pandemic.

#### Wave 6: Resilience and coping strategies

We analysed people’s coping and their coping strategies during the pandemic using the survey data collected at wave 6 (corresponding to the time of Focus Group 3). We held ‘wave 6’ survey data collection in August 2020 (upon first UK Lockdown being fully lifted) with a total of 4584 participants. (The Table 6 below shows the demographic characteristics of the participants).

**Table 6:** Descriptive statistics of participants at wave 6

Participant characteristics	Category	N	Percent
Gender	Female	2,461	53.69
	Male	2,123	46.31
Age group	18–24	425	9.27
	25–34	709	15.47
	35–44	756	16.49
	45–54	768	16.75
	55 +	1,926	42.02
Social grade	C2DE, working class	1,825	39.81
	ABC1, middle class	2,759	60.19
Work status	Working full time	1,719	37.5
	Working part time	666	14.53
	Full time student	197	4.3
	Retired	1,214	26.48
	Unemployed	242	5.28
	Not working/Other	546	11.91
Marital status	Married/ Civil Partnership	2,138	46.64
	Living as married	600	13.09
	Separated/ Divorced	400	8.73
	Widowed	191	4.17
	Never Married	1,237	26.99
		18	0.39
Country	England	3,830	83.55
	Wales	237	5.17
	Scotland	404	8.81
	Northern Ireland	113	2.47

**Fig. 3:**
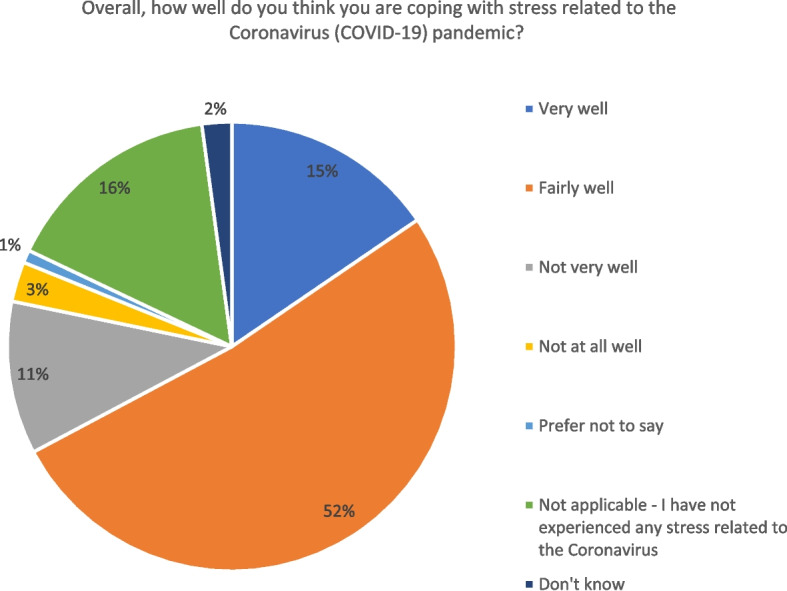
Coping (overall situation)

During survey data collection wave 6, we asked participants the question: “Overall, how well do you think you are coping with stress related to the Coronavirus (COVID-19) pandemic?” As shown in Fig. 3 below, among a total of 4584 participants, around 16% of people indicated that they have not experienced any stress, 15% said they were coping well, 52% expressed they were coping fairly well, 11% were not coping very well, and 3% expressed that they were not coping well at all.

**Fig. 4:**
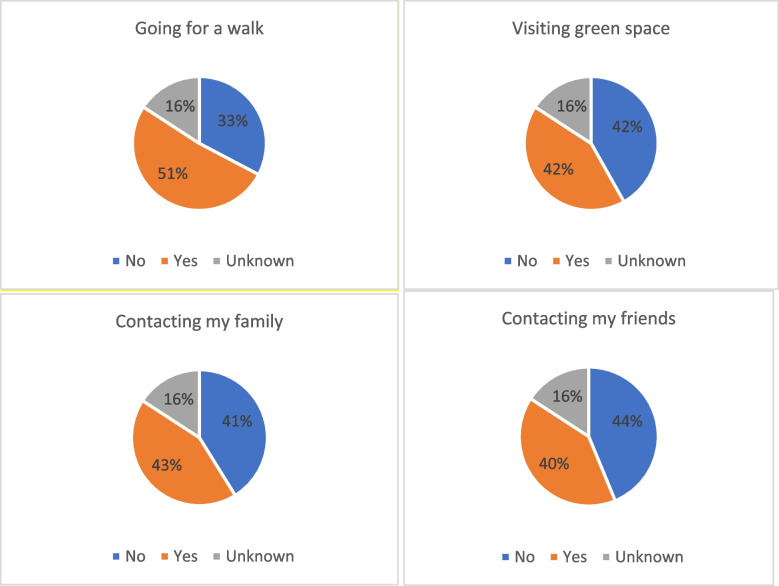
Coping strategies adopted (overall situation)

In terms of coping strategies, we asked participants the question: “Which, if any, of the following have helped you to cope with stress related to the Coronavirus (COVID-19) pandemic in the past 2 weeks?” Out of a series of coping strategies, accessing nature and contacting family and friends proved very helpful. As shown in Fig. 4 below, among a total of 4584 participants, around 51% expressed that going for a walk outside was helpful for coping with stress during the pandemic, 42% said being able to visit green spaces (e.g. outdoor spaces, parks, etc.) was a useful coping strategy, 43% expressed that contacting my family (e.g. phone, video chat, etc.) was helpful for coping, and 40% said contacting my friends (e.g. phone, video chat, etc.) was useful.

Subsequently, we conducted the third FGD tailored around the topic “Resilience and coping strategies” based on the wave 6 survey data findings, which revolved around people’s resilience and coping strategies during the pandemic, as further reported below.

### Focus group discussions results

The emerging findings of FGDs 1 and 2 were mainly focused around current experiences and emotional challenges, while those of FGD 3 were more focused around resilience, coping strategies and ways forward. Table 7 presents a short summary of the main findings of each FGD.

**Table 7:** Key messages from focus group discussions

**FGD 1: Socio-economic inequalities and mental health**
Participants stressed the fact that, although UK lockdown measures were applied across the country, not everyone experienced its consequences in the same way. Participants expressed the need to view overall health, wellbeing, and financial security as equally important
**FGD 2: Diverging inequalities and mental health experiences**
Participants discussed how people entered the pandemic differently from positions of advantage/disadvantage. Certain population groups in our society already had a higher risk of experiencing poor mental health and wellbeing than people from more advantaged positions, and more nuanced views were identified in relation to financial disparities, its consequences and impacts on mental health
**FGD 3: Resilience and coping strategies**
Participants described their main coping strategies to be accessing nature, working from home, and maintaining relationships with family and friends

Three broad overarching themes were identified, each with several sub-themes (Table 8). The data are presented in the form of a summary of key themes, evidenced with illustrative quotes. Quotes indicate the workshop (numbered FGD 1 to FGD 3) and include a unique participant identifier.

**Table 8:** Overview of main themes and sub-themes

**A. Impacts of the pandemic**
1. Changing and widening socio-economic inequalities
2. Emotional impact
**B. Moving forward: needs and recommendations**
1. Support needs
2. Reopening of society
**C. Coping mechanisms and resilience**
1. Accessing nature
2. Working from home
3. Connecting with others

#### A. Impacts of the pandemic

##### 1. Changing and widening socio-economic inequalities

In all FGDs, participants agreed that, whilst we are all going through the pandemic and related restrictive measures, it is affecting everyone in different ways, depending on their age, demographic background, employment sector, job type and contract, geographical area, belonging to an at-risk group and more. Self-employed people, small businesses, young people, people with disabilities, people from ethnic minorities, domestic abuse survivors, people who are still paying off debts, informal carers, parents managing home-schooling and many more were considered high risk groups in the context of our discussions around ‘inequalities and financial impacts on mental health’. As a participant said:

“I see almost a kind of big matrix, where you have, sort of the effects on the population as a whole. And then kind of additional effects at different times, for different groups. And then you begin to build up the picture of how it affects the population as a whole.” (male, p3, GoFGD2).

Participants voiced quite strongly that inequalities have emerged during the pandemic and were going to widen between those in different job sectors and with different job conditions; in FGD2 the same participant reported that:

“I think the inequalities have really changed as a result of the pandemic. At one end we have a lot of people who had steady jobs, and have suddenly found that they’ve got no income. It might be two adults who have lost their income totally, at least in the short term. And although there are things like Universal Credit, and so on, to get hold of, it's not easy getting those in time, or getting the loans they need to put bread on the table in the short term, never mind keeping the mortgage going, or having to deal with the back payments. And on the other hand, there are people I can think of who have been lucky enough to be allowed to work at home for the first time.” (female, p11, GoFGD2).

Participants expressed worries that other factors – such as the UK leaving the European Union – might further exacerbate financial insecurity and inequalities. A participant reported:

“We've got Brexit that’s still in the background, and how is that going to affect the price of food, the availability of food? Personally, I need fresh green, it's very helpful for my condition. Where is the guarantee that the prices of simple food stuff isn't going to go through the roof?” (male, p24, GFGD1).

As restrictions continued, participants’ worries increased and in FGD2 were accompanied by a sense of powerlessness, and perceived lack of guidance from the government:

“We’re seeing massive inequality, because everything has been structurally laid out to make sure it ends up that way. And how do you feel in that, you feel powerless.” (female, p11, PFGD2).

Moving into lockdown, more concerns were expressed for children and young people at various stages of transition, such as those who were not able to complete exams and were moving to secondary school, college, university, apprenticeships and first jobs. The same young person attending our FGDs highlighted their sense of worry and loss and said:

“I do feel like this has been quite triggering in regards to my frustration and resentment towards the job market, and going up the job market. […] And I'm a first-generation migrant, so since my family has arrived in this country, I've been trying my best to work up the socio-economic ladder. And I feel like this pandemic, as we are all aware, is exacerbating existing inequalities, and existing forms of oppression. And I feel like, I can probably understand why young people feel anxious, when it feels like their whole, like, their whole generation is being wasted.” (p10, PFGD2).

##### 2. Emotional impact

Worries and emotional concerns moved in a continuum, ranging from the fulfilment of basic everyday needs (e.g. being unable to find supplies from the supermarket, having heating in their households) to an increased uncertainty about the future. Lack of security was often linked to the unpredictable nature of the situation:

“…we can't visualise what is in our future, at the moment, we don't know what we’re moving to, we don't know what that looks like. And especially if our identities are tied to what we do, and that’s changing. […] So we don't know where we’re going, and that’s really uncertain…” (female, p31, NFGD2).

Feelings of isolation and a desire to be able to choose whether to be with others or not were often mentioned and had many nuances. Specific issues were raised around older people and loneliness, with reference to this having already pre-existed the pandemic. For others, the experience of loneliness during the pandemic related to living in the UK, away from family abroad:

“As someone who is not British, I have family in a different country who I haven’t been able to see in a long time. It's my birthday in a few days, which I'm going to be celebrating alone. So I think we can also look at, like, these experiences of people who are in the UK alone, people who have family elsewhere.” (male, p15, EFGD2).

Some participants noticed that organisations that run mental health support helplines were reporting a higher percentage of first-time callers, thus suggesting that the pandemic and related measures were also having an impact on those without any pre-existing mental health conditions:

“Lots of people who don't have pre-existing conditions, who haven’t previously thought they needed help with mental health, or wellbeing, however you want to put it, are now for the first time saying, yeah actually, I've got an issue here. So there is that kind of population level, you know, anxiety, as a feeling.” (male, p12, GoFGD2).

#### B. Moving Forward: needs and recommendations

##### 1. Support needs: what needs to be done

Participants identified and demanded the need more funding to address the social determinants of health and wellbeing, arguing that the huge social and structural inequalities and issues are beyond the individual’s grasp and necessitate a whole-systems response and holistic approach:

“As you might see more people in sort of mental health crisis, where it's been really quite seen as medicalised, or as a disorder, when in fact, it's really about their financial situation that they have been put in. So, I think there's something about it being on top of lots of other things that people are already coping with. And that I think it'll be hard, but I think it's quite important to stay quite focused on social causes of the crisis and difficulty at this time; few people focused on the social determinants of the current mental health crisis.” (female, p5, NaFGD1).

The same participant then highlighted the key role the voluntary sector plays in supporting communities:

“I think it's worth remembering that, like, within charities, we’re all humans, too, and a lot of the charities are paralysed because of the sheer number of demands upon them. So, in terms of what Government and large charities can do, it's actually supporting that specialist work, to make the community-based stuff happen. Otherwise, yeah, a lot of really small specialist places are going to go, I think, unfortunately.” (female, p5, NaFGD1).

In FGD2, more issues were identified around communication, information-sharing and transparency across the different jurisdictions of the UK (England, Scotland, Wales and Northern Ireland) in relation to lockdown, to the lifting of restrictions and to physically going back to work. This generated confusion, stress and anxiety about who to believe and about who to follow:

“It's worth noting devolution. So, in Wales, we’re a few weeks behind what you're doing in England. Scotland, again, slightly different. And I think it's difficult for people to know where they should get their information from.” (female, p4, NFGD2).

Further requests for clarity were made around the amount and length of Government financial support available (e.g. the Government’s furlough scheme and financial support grants), ongoing issues with Universal Credit, self-employment, the challenges of the job market, and the implications for those who were shielding, with people expressing a sense of uncertainty about their future:

“Eventually the Government, the financial support is going to go, it'll taper off and it'll stop, and that is going to lead to uncertainty for people who don't know whether they have a job to go back to. Or if you're self-employed and your business isn't recovering, to bring in the income that you need. A lot of people have had huge issues with Universal Credit, in terms of the application process, but then also what that’s meant for their income and other benefits.” (female, p13, NaFGD2).

##### 2. Re-opening of society

Experiences related to meeting up again varied across participants. A few mentioned they either enjoyed not having to meet up in big groups or would have felt anxious meeting because they were shielding. Some felt growing expectations to meet up upon lifting of the lockdown, especially for shielding people, at-risk population groups such as people belonging to ethnic minority communities at higher risk of COVID-19, and people with long-term health conditions:

“In my household, I've got my mum here who's 70, and my husband who is Indian, so they're both kind of higher risk, and we’re still acting as if we’re in lockdown. But then, we’re getting some level of social pushback of, like, why don't you want to meet? Why don't you want to do this? […]. So I think especially for people who have been told to shield, and then they're suddenly being told not to, if that was me, I wouldn’t have the confidence that it was suddenly okay, you know, what’s changed…”. (female, p4, NFGD2).

According to our participants, for a minority of children online education was working better than face-to-face, and they will need extra support once they return to school:

“A number of young people who perhaps have social and communication difficulties, who are finding online and home-schooling easier, and are going to struggle going back into mainstream school.” (female, p13, NaFGD2).

Participants emphasised the types of strain felt by people in later life due to lockdown measures and how lifting the restrictions may have caused a new worry that they would no longer benefit from the same level of protection as during full lockdown. Some had a perception that the end of the lockdown was pushed by the demands of those of working age, most of whom do not have the same health risks as the older population:

“There's two million people that have been shielded, […] Of those, the vast majority are over 60. Those people are not satisfied […] with the public health measures which have been put into place to entice them out of their homes. […]. Their support is going to be cut off. And all these people are extremely concerned about life and death, and that is, you know, a very big issue.” (male, p6, DFGD2).

Concerns were expressed in relation to health and safety at work, crowded public transport when commuting to work and being in the workplace:

“Those who have jobs that are more manual or who have to work face-to-face will have a different and more difficult experience and face more challenges than those who can work from home.” (male, p6, EFGD2).

#### C. Coping mechanisms and resilience

##### 1. Accessing nature

Participants agreed that the allowance of one daily outdoor form of exercise during lockdown was very beneficial and a great help with coping. They particularly valued being able to go for a walk and connecting with nature, which was the main reported strategies that people used to support their mental health:

“Gardening and walking the dogs were really key for me, and during the early phases when we weren’t allowed to go very far. I tend to go out to the coast most weekends if I can, and go for good long walks and look at the sea, and I found that really difficult. And actually, when we were…the first time you were allowed to do that, I actually felt quite tearful just being there and just feeling that sort of, you know, nature around me, so yeah, that was a big part of it for me.” (female, p14, JAFGD3).

Even those who couldn’t travel far found a way to benefit from connecting with nature:

“I have a balcony, so I think a couple of months in, I invested in some plants and I started to garden, and I felt like definitely when the weather was nice, it would just be like a space for me to kind of escape to and really bring me back into the present.” (female, p11, VFGD3).

##### 2. Working from home

In all three FGDs, participants described the significant benefits of working from home in terms of impact on mental health, fatigue, family and finances. Working from home was perceived as one of the biggest benefits, and there were suggestions to introduce a ‘flexible working rights’ law:

“People who are in regular employment are now working from home, and I think that does come with certain financial benefits, like not having to commute, not having to buy food outside. And as someone with mental health disabilities, that does have a positive effect on my mental health, but I also realise that that’s a privilege, and not everyone will be in the same stable employment situation like I am.” (female, p20, VFGD1).

Some people felt protected at home from the worries of meeting other people when not ready:

“I’ve been working from home, which I quite like, but the only difference is, there’s no kind of pressure to meet up with anybody, no-one’s kind of pressuring me to go out, […]. I think I’d probably feel more stressed if I was sort of being forced into contact with people, and I wonder how people who aren’t living with others or going into work are feeling about that, like if they’re getting pressure from people to meet up maybe when they’re not ready, or if that’s adding stress.” (female, p7 SFGD3).

##### 3. Connecting with others

Online presence and contact with family and friends was reported by most as very helpful, although experiences were individualised and multi-faceted. Most participants of FGD3 were feeling comfortable with the person/people in their own household and/or social media contacts. However, they identified heightened worry, stress and anxiety about meeting up outside their home, especially in high-risk places (e.g. crowded spaces) and with ‘reckless’ people (e.g. those who did not follow the rules of physical distancing), whilst meeting up in a park or big open space where there are few people was seen as less worrying:

“I am living with my parents, and I have my friendship groups. I also have professional contacts and – since lockdown lifted – I occasionally am meeting up with others. Yes, social contacts with others keep me grounded.” (female, p 16, PFGD3).

Community connectedness has been a crosscutting theme, the place where people live and come together to support each other played a central role, as highlighted, for example, by some people in FGD1:

“I started volunteering in my neighbourhood as well, because I felt less powerless, I was feeling so powerless, and I was thinking, there’s nothing I can do, so getting involved in the mutual aid groups was really helpful, because I thought, okay, at least there’s something I can do and get back on track.” (female, p17, SaFG3).

## Discussion

The present mixed-methods research described UK adults’ experiences and perceptions of inequalities and related mental health impacts during the Coronavirus pandemic as well as the main coping strategies people employed to support their mental health. 

As evidenced by the findings from this inequalities sub-study as well as those of our wider ‘Mental Health in the Pandemic’ study, the pandemic has exacerbated existing inequalities, created new ones and revealed critical societal needs as well as strengths [2, 11, 12, 29, 30].

Results highlighted how public health restrictions introduced to limit the spread of COVID-19 had an impact on the mental health of many. One-fifth of the surveyed UK population had experienced a sustained increase in poor mental health by September 2020. Rates of anxiety and depression were particularly high during periods when the tightest physical-distancing restrictions were in place. Those facing financial hardship fared worse than others [3, 22]. These results prompted qualitative data gathering through FGs discussion, in order to gain a deeper understanding of individual experiences, perceptions and coping strategies, and how these evolved during the pandemic.

When discussed with the participants of our FGD, both the perceived social pressures (e.g. to go back to work) and the perceived lack of clear evidence-based information from the UK government as were identified a potential cause of stress. Many reported low confidence in leaving the house and meeting others or going back to work. Aknin et al. (2022) have highlighted how stricter pandemic policy measures are associated with slightly worse mental health impacts [31]. Furthermore, government policies leading to a loss in social connection and primarily adopted in ‘mitigator’ countries (where restrictions on gatherings and stay-at-home requirements were applied) have been associated with greater psychological distress and lower life evaluations.

Participants expressed significant concerns around to the unequal socio-economic positions from which people faced the pandemic and the new inequalities that have been arising and widening in society (e.g. between rich and poor, in different job sectors, different job conditions, different housing and environments, access to safe green spaces, digital connectivity, and more). The pandemic has had a significant impact on the economy and health, especially for the most vulnerable social groups, with some being more affected than others [30, 32].

Participants highlighted that, for the majority of them, working from home was a great help but acknowledged that not all could benefit from working from home. Often people in more deprived communities live in inadequate housing not conducive to working from home, and are also more likely to have to work away from home, travel by public transport and work in roles associated with higher exposure to COVID-19 (e.g. security guards, public transport workers, taxi drivers, retail employees, health and social care workers, cleaners and hospitality staff) [16, 33]. Furthermore, low rates and coverage of statutory sick pay, and difficulty in accessing isolation payments reduced people’s ability to self-isolate and increased their exposure to SARS-CoV-2 and develop COVID-19 [34].

Our findings illustrated how these disproportionate effects impacted people's experience of the pandemic. Inequalities in relation to the social determinants of health are one of the most relevant risks for becoming infected and developing COVID-19, due to the health consequences for those who are exposed to it [35]. There are inequalities in COVID-19 morbidity and mortality rates, reflecting existing unequal experiences of chronic diseases and the social determinants of health, as often happens during pandemics [36]. The lack of economic resources is an indicator of social determinants of health in vulnerable populations, due to having to accept more precarious or insecure jobs and worse living conditions [35]. The Institute for Fiscal Studies (2021) revealed that public health measures reduced the ability of many to work. However, job-support schemes meant that disposable income inequality fell. In the longer term, however, lower work experience for the less educated and missed schooling could push up some inequalities. Increased rates of working from home will probably stay, which may potentially increase some inequalities but decrease others. Furthermore, flexible working arrangements (such as working from home, hybrid working, part-time work, job-sharing, compressed hours, and more) have helped open up the job market to many who previously would have been excluded from it (e.g. people for whom commuting is very difficult or impossible due to caring responsibilities, fluctuating health conditions, certain disabilities, or those who cannot afford expensive commutes, expensive childcare cover, and more). These people were previously excluded from a large part of the job market and the benefits of being employed, whereas much more flexible working arrangements has enabled them to be part of the working world [37]. At the same time, with people being enabled to work more from home and spending more time in their local communities, our participants reported benefits such as improved work-life balance and family-life, improved social connections and opportunities in local communities, more local shops and start-ups (which contributes to reviving and bringing together local communities), more emphasis on local services and infrastructure (including safe outdoor spaces and parks), all of which should be encouraged by the UK Government as part of the ‘Levelling Up’ agenda (the ‘Levelling Up’ agenda is a moral, social and economic programme for the whole UK Government aiming to spread opportunities and prosperity more equally across the UK) [38].

Additionally, the current study uncovered participants’ suggestions concerning support in moving forward through the pandemic and beyond. They highlighted how more financial support was needed in regard to addressing the social determinants of mental health and they recommended a whole system preventative response. Marmot et al. [39] highlighted that the COVID-19 pandemic amplifies existing inequalities in society. This requires the implementation of coordinated measures of prevention, diagnosis, and treatment of the pandemic, with the aim to avoid an increase in inequality as well as the identification of vulnerable groups who require more economic assistance to recover from the pandemic [16]. From the FGDs it also emerged that the third sector played a key role in supporting individuals and communities and in coordinating some of those measures. Since the beginning of lockdown restrictions, there has been a 12% increase in calls and their duration to the Samaritans [40] (often the last resort charity for people in distress), as also mentioned in our FGDs. It is imperative that the voluntary sector, which provides an essential supporting role to the state when it comes to mental health, remains afloat and funded. The voluntary sector is crucial in the Coronavirus response because it supports and safeguards large parts of the population, and it is part of that multi-systemic approach that can help build resilience beyond the health system's response to COVID-19 [41, 42]. The level of financial and other support from the government should reflect that. Any government support should be equitably distributed and should safeguard the diversity of the sector [12].

The majority of our FGD participants mentioned that spending time outdoors and connecting with nature was one of their main coping strategies. Our ‘Mental Health in the Pandemic’ study shows that 59% of people went for a walk outside and 50% have been able to visit green spaces as a way of coping with the stress of the pandemic [29]. Between April and June 2020, fewer than half of adults reported they were spending more time outside, but three quarters reported they were noticing and engaging more with everyday nature [43]. These changes in the relationship with nature contributed to improvements in people’s wellbeing, particularly in feelings of life being worthwhile [44]. However, natural spaces are currently not equally accessible to all and maybe particularly inaccessible to certain groups because of other social, economic and health inequalities. Those in deprived communities have worse access to high-quality and safe green spaces which, as we move out of the pandemic, is likely to lead to more crowded meetings in some outdoor places or more meetings in higher risk indoor spaces [45–47]. Mental health benefits from connecting with nature may vary by socioeconomic status, residential location, occupation, disability, culture, gender, and age. Some places are inaccessible to older people with limited physically mobility [44]. It is recommended that innovative solutions are implemented to facilitate access of green environments to an ageing population and to make our towns accessible and inclusive [48].

Social contact was also a valuable way of coping with the stress of the pandemic, with differences in the types of daily contact that people felt comfortable with. Understanding the ways in which policymakers can balance physical health and psychological health while managing physical distancing has generated recent interest [49, 50]. Striking this balance is crucial, as physical distancing for extended periods of time may strain people’s needs for social connection to such an extent that they may eventually disregard policy guidelines [51]. Therefore, learning from this and other pandemics and epidemics is crucial in gaining a deep understanding of the challenges—including the mental health challenges—people face during such time, how best to prevent (if/where possible) and address these, and to be as best as possible prepared for any future eventualities.

## Strengths and limitations

This mixed-methods study has several strengths. To the best of our knowledge, this is one of the first mix-methods studies exploring UK adults’ experiences and perceptions of inequalities on mental health linked to the pandemic. The quantitative findings from our UK-wide representative cross-sectional survey with a large sample size have better informed our Focus Group Discussion topic guides. Informed by our cross-sectional surveys, the multiple FGDs across time enabled us to capture changes during the early phases of the pandemic. The FGDs provided in-depth knowledge of people’s experiences and views during the pandemic and also provided much needed depth, context, understanding and meaning to the cross-sectional survey findings. Our literature reviews and community conversations as part of our ‘Mental Health in the Pandemic’ study have also provided rich contextualisation for our study [10].

There were also some limitations. This study adopted a repeated cross-sectional approach where sample population were different at each wave. Caution is needed to interpret changes over time and between-subject variability across waves needed to be considered. Digital poverty (particularly for the older population) and the digital divide may hamper representativeness and data validity of online surveys. Other limitations include the usual caveats of using self-reported data in surveys.

## Conclusion

Whilst the quantitative cross-sectional survey data highlighted a range of worries (e.g. financial concerns, losing own job), different emotional experiences (e.g. loneliness, anxiety) and a series of coping strategies (e.g. spending time in nature, connecting with family and friends); the qualitative Focus Group Discussions helped to gain a deeper understanding and contextualisation of those experiences and how inequalities unfolded. Participants validated the view that protecting people’s mental health in the context of a wider health crisis is of critical importance, and concerns were raised about emerging inequalities. Participants saw addressing health inequalities as an important part of the government’s role in recovery. Their experiences show a clear need for policies that valued and supported a more holistic view of mental health, addressing infrastructural and systemic issues such as work, income, education, good access to digital tools, and housing. Therefore, a public health approach to mental health would be more suitable to address both social determinants and medical needs [52]. Participants acknowledged that there will be a need for targeted solutions for the inequalities groups coming out of lockdown. This includes adequate support is needed on a longer-term basis to prevent the financial strain that strongly risks poor mental health. While short-term ad-hoc support to mitigate the risks and effects of the pandemic partially alleviated economic problems for many, the persistent structural issues mentioned above seem to have impacted people’s ability to cope. There is a need for action to reduce those existing social and economic inequalities when planning for both the recovery and future pandemic phases.

### Supplementary Information

**Additional file 1.**

**Additional file 2.**

**Additional file 3.**

## Data Availability

We intend to make our survey data available upon completion of the overall ‘Mental Health in the Pandemic’ study and dissemination of all our main findings. The platform through which we aim to do this is DATAMIND (Health Data Research UK): https://www.hdruk.ac.uk/helping-with-health-data/health-data-research-hubs/datamind/ Please contact Professor Tine Van Bortel regarding availability of data and materials.

## Footnotes

1 On July 4th 2020, the Government announced a relaxation in national lockdown rules, which included reducing physical distancing measures, permitting separate households to meet indoors, allowing restaurants and businesses to reopen and people who had been shielding were able to spend time outdoors in a group of up to six people.
