# Supplementary material for: Inequalities and mental health during the Coronavirus pandemic in the UK: a mixed-methods exploration
Source: BMC Public Health. 2023 Sep 20;23:1830. doi: 10.1186/s12889-023-16523-9 (PMC10510114; doi:10.1186/s12889-023-16523-9)
Supplement: Supplementary file 1 — Additional file 1. [file 12889_2023_16523_MOESM1_ESM.docx]

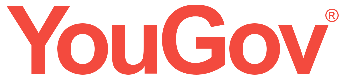


YouGov Omnibus Standard Order Form UK Omnibus

Order: Nationwide UK Omnibus

**QUESTIONNAIRE**

#PAGE 1

*Base: All UK Adults 18+*
*Question type:* ***Pdl***
*#Question display logic:*
***if pdl.profile_work_stat.last > months(9) and updated***
*[Varlabel - Employment Status Main]*

**[profile_work_stat]** Which of these applies to you?

| <1> | Working full time (30 or more hours per week) |
| --- | --- |
| <2> | Working part time (8-29 hours a week) |
| <3> | Working part time (Less than 8 hours a week) |
| <4> | Full time student |
| <5> | Retired |
| <6> | Unemployed |
| <7> | Not working |
| <8> | Other |

#PAGE 2

*Base: All UK Adults 18+*
*Question type:* ***Multiple***
*#row order: randomize*
*[Varlabel - How you have been affected by COVID19 in past 2 weeks]*

**[LMC_QA]**

**The 2019 novel coronavirus (2019-nCoV), otherwise known as COVID-19, is an infectious disease first identified in the city of Wuhan, capital of Hubei province in China, after 41 people developed pneumonia without a clear cause. Infections have since been reported around the world. Symptoms include fever, coughing and breathing difficulties.**

**Throughout this questionnaire, we will refer to the disease as Coronavirus.**

**For the following question, we would like you to think about yourself, members of your household or other family members who have been affected by Coronavirus.**

Which, if any, of the following applies to how you have been affected by Coronavirus (COVID-19) in the **past 2 weeks**? (Please select all that apply)

| <1> | I have been tested for Coronavirus and had a positive result |
| --- | --- |
| <10> | I have been tested for Coronavirus and had a negative result |
| <2> | Someone in my household has tested positive for Coronavirus |
| <3> | A family member living at a different address has tested positive for Coronavirus |
| <4> | I am self-isolating with symptoms of Coronavirus |
| <5> | My household is self-isolating because someone else has symptoms of Coronavirus |
| <6> | A family member living at a different address is self-isolating with symptoms of Coronavirus |
| <7> | As part of my current work I am working directly with individuals who have tested positive for Coronavirus |
| <966 fixed xor> | None of these |
| <977 fixed xor> | Don’t know |

*#option display logic:*
***<7> - If [profile_work_stat] - Working full time (30 or more hours per week), Working part time (8-29 hours a week), Working part time (Less than 8 hours a week), is selected [if profile_work_stat in [1,2,3]]***

#PAGE 3

*Base: All UK Adults 18+*
*Question type:* ***Multiple***
*#row order: randomize*
*[Varlabel - Agreement with statements regarding Coronavirus]*

**[LMC_Q1]** Which, if any, of the following statements do you agree with regarding Coronavirus (COVD-19 in the **past 2 weeks**? (Please select all that apply)

| <1> | I feel well informed about the need for measures to control the spread of the outbreak personally |
| --- | --- |
| <2> | I have everything I need to manage if I am self-isolated (e.g. sufficient food. etc.) |
| <3> | I understand the benefit of washing my hands often |
| <7> | I understand the benefit of self-isolating if needed to |
| <4> | I feel I can make a positive contribution to efforts to limit the spread/impact of the outbreak |
| <966 fixed xor> | None of these |
| <977 fixed xor> | Don’t know |

#PAGE 4

*Base: All UK Adults 18+*
*Question type:* ***Multiple***
*#row order: randomize*
*[Varlabel - Emotions as a result of Coronavirus]*

**[LMC_Q2] For the following few questions, we have provided you with a "Prefer not to say" option, which you can select if you do not wish to share your experiences on a particular question.**

Which, if any, of the following emotions have you felt as a result of the Coronavirus (COVID-19) pandemic in the **past 2 weeks**? (Please select all that apply)

| <1> | Afraid | <8> | Ashamed |
| --- | --- | --- | --- |
| <2> | Panicked | <9> | Guilt |
| <3> | Anxious or worried | <10> | Loneliness |
| <4> | Empathetic | <11> | Unprepared |
| <5> | Indifferent | <966 fixed xor> | None of these |
| <6> | Hopeful | <977 fixed xor> | Don’t know |
| <7> | Hopeless | <933 fixed xor> | Prefer not to say |

#PAGE 5

*Base: All UK Adults 18+*
*Question type:* ***Grid***
*#row order: randomize #max number of choices per page: 5*
*[Varlabel - Understanding of how worried people are about Coronavirus]*

**[LMC_Q3] For the following question, we have provided you with both a "Not applicable" and "Prefer not to say" options, as some questions may either not apply or are of a sensitive nature. Please select the option that best applies to you.**

Have you been worried about any of the following as a result of the Coronavirus (COVID-19) pandemic in the **past 2 weeks**? (Please select one option on each row)

| -[LMC_Q3_1] | Financial concerns (e.g. going into debt, ability to pay bills, etc.) | -[LMC_Q3_10] | Having enough food to meet my / my households basic needs |
| --- | --- | --- | --- |
| -[LMC_Q3_2] | Losing my job | -[LMC_Q3_11] | Being vulnerable because of an existing medical condition |
| -[LMC_Q3_3] | Becoming ill with the virus | -[LMC_Q3_12] | My education or career training being interrupted |
| -[LMC_Q3_4] | Being able to cope with uncertainty (e.g. not knowing what will happen) | -[LMC_Q3_13] | Looking after my children if I get sick |
| -[LMC_Q3_5] | Having no-one to care for me, as a result of becoming ill with the virus | -[LMC_Q3_14] | Passing the coronavirus on to someone else if I became infected |
| -[LMC_Q3_6] | Being unable to access my benefit payments | -[LMC_Q3_15] | Being vulnerable because of my age |
| -[LMC_Q3_7] | Not being able to care for friends and family, as a result of becoming ill | -[LMC_Q3_16] | Experiencing discrimination if I contract the Coronavirus |
| -[LMC_Q3_8] | Being separated from friends and family | -[LMC_Q3_17] | Making my existing mental health problems worse |
| -[LMC_Q3_9] | Being able to cope with self-isolation | -[LMC_Q3_18] | How the mental health of my child(ren) will be affected by the pandemic |

| <1> | Yes |
| --- | --- |
| <2> | No |
| <3> | Don't know |
| <944 fixed xor> | Not applicable |
| <933 fixed xor> | Prefer not to say |

#PAGE 6

*Base: All UK Adults 18+*
*Question type:* ***Multiple***
*#row order: randomize*
*[Varlabel - Statements that apply with regard to Coronavirus]*

**[LMC_Q4] Still thinking about the Coronavirus...**

In the **past 2 weeks**, which, if any, of the following statements apply to you? (Please select all that apply)

| <1> | I would volunteer to help tackle the Coronavirus as long as it didn’t increase my risk of catching it (e.g. dropping food off to people's houses, etc.) | <8> | I believe that concern about the Coronavirus is exaggerated |
| --- | --- | --- | --- |
| <2> | I would volunteer to help tackle the Coronavirus even if it increased my personal risk of catching it (e.g. dropping off food to people's houses, caring for people who are sick. etc.) | <9> | It has negatively affected how well I sleep |
| <3> | I am worried about someone I know who is living alone and may need help during the pandemic | <10> | I am caring for someone and worried about how they will cope if I became infected |
| <4> | I am planning to help someone I know who is living alone during the pandemic | <11> | I am a carer and worried about how I will cope if I became infected |
| <5> | I am worried about someone who is particularly vulnerable (e.g. elderly, someone with a pre-existing medical condition) | <966 fixed xor> | None of these |
| <6> | I live alone and will not have support of family or friends if I became infected | <977 fixed xor> | Don’t know |
| <7> | I think tackling the Coronavirus is solely the job of Government, public services and NHS | <933 fixed xor> | Prefer not to say |

#PAGE 7

*Base: All UK Adults 18+*
*Question type:* ***Single***
*[Varlabel - How well are people coping with stress of COVID19]*

**[LMC_Q5] For the following question, if you have not experienced any stress related to the Coronavirus pandemic, please select the 'Not applicable' option.**

Overall, how well do you think you are coping with stress related to the Coronavirus (COVID-19) pandemic?

| <1> | Very well |
| --- | --- |
| <2> | Fairly well |
| <4> | Not very well |
| <5> | Not at all well |
| <977 fixed xor> | Don’t know |
| <933 fixed xor> | Prefer not to say |
| <944 fixed xor> | Not applicable - I have not experienced any stress related to the Coronavirus |

#PAGE 8

*Base: All UK Adults 18+ who have experienced stress related to the Coronavirus pandemic*
*Question type:* ***Multiple***
*#row order: randomize*
*#Question display logic:*
***If [LMC_Q5] - Very well or Fairly well or Not very well or Not at all well or Don’t know or Prefer not to say, is selected [if LMC_Q5 in [1,2,4,5,977,933]]***
*[Varlabel - Helped with stress related to the Coronavirus pandemic]*

**[LMC_Q6] For the following question, if nothing has helped you cope with stress related to the Coronavirus pandemic, please select the 'Not applicable' option.**

Which, if any, of the following have helped you to cope with stress related to the Coronavirus (COVID-19) pandemic in the **past 2 weeks**? (Please select all that apply)

| <1> | Contacting my family (e.g. phone, video chat, etc.) | <8> | Contacting a support group (i.e.. where members with the same issues can come together for sharing coping strategies, to feel more empowered and for a sense of community) |
| --- | --- | --- | --- |
| <2> | Contacting my friends (e.g. phone, video chat, etc.) | <9> | Going for a walk outside |
| <3> | Contacting a mental health worker or counsellor (e.g. via phone, video chat etc.) | <10> | Doing a hobby |
| <4> | Maintaining a healthy lifestyle (e.g. balanced diet, enough sleep, exercise etc.) | <11> | Volunteering to help in my local community or for the NHS |
| <5> | Keeping up to date with relevant information (e.g. TV news, newspapers, online etc.) | <955 fixed> | Other [open] please specify |
| <6> | Limiting my exposure to the news about the Coronavirus | <977 fixed xor> | Don’t know |
| <7> | Limiting exposure to social media (e.g. Facebook, Instagram, Snapchat, Twitter etc.) | <933 fixed xor> | Not applicable - Nothing has helped me to cope with stress related to the Coronavirus |

#PAGE 9

*Base: All UK Adults 18+*
*Question type:* ***Grid***
*#row order: randomize*
*[Varlabel - What done more or less of in the past 2 weeks]*

**[LMC_Q7] For the following question, if you do not normally do any of the following, please select the 'Not applicable' option.**

**We have also provided you a 'Prefer not to say' option as the question might be of a sensitive nature.**

Which, if any, of the following have you done more or less of to cope with the stress of the Coronavirus (COVID-19) pandemic situation in the **past 2 weeks,** or has there been no change? (Please select one option on each row)

| -[LMC_Q7_1] | Drunk alcohol |
| --- | --- |
| -[LMC_Q7_2] | Smoking (e.g. cigarettes, cigars, etc.) |
| -[LMC_Q7_3] | Used illicit drugs or other substances |
| -[LMC_Q7_4] | Eaten too much |
| -[LMC_Q7_5] | Eaten too little |

| <1> | More |
| --- | --- |
| <2> | Less |
| <3> | No change |
| <944 fixed xor> | Not applicable |
| <933 fixed xor> | Prefer not to say |

#PAGE 10

*Base: All UK Adults 18+*
*Question type:* ***Single***
*[Varlabel - Consent to continue with sensitive topic questions]*

**[LBC_Q8A] The following questions are on the topic of self harm and suicidal thoughts, and your own personal experiences of this. We understand this can be a sensitive topic, but please remember your answers will always be treated anonymously and will never be analysed individually.
We will provide you with a "Prefer not to say" option for particularly sensitive questions, which you can select if you do not wish to share your opinion or experiences on a particular question.**

Are you happy to continue with this section of the survey?

| <1> | Yes |
| --- | --- |
| <2> | No |

#PAGE 11

*Base: All UK Adults 18+*
*Question type:* ***Dyngrid***
*#row order: randomize*
*#Question display logic:*
***If [LBC_Q8A] - Yes is selected [if LBC_Q8A == 1]***
*[Varlabel - Experiences as a result of the Coronavirus pandemic]*

**[LMC_Q8B]** Have you done or experienced any of the following, as a result of the Coronavirus (COVID-19) pandemic in the **past 2 weeks**? (Please select one option on each row)

| -[LMC_Q8_1] | Experienced suicidal thoughts/ feelings |
| --- | --- |
| -[LMC_Q8_2] | Deliberately hurt myself |
| -[LMC_Q8_3] | Worried about someone close to me dying |

| <1> | Yes |
| --- | --- |
| <2> | No |
| <977 fixed xor> | Prefer not to say |

#PAGE 12

*Base: All UK Adults 18+ who have done or experienced suicidal thoughts, self harm or worried about someone dying in the past 2 weeks*
*Question type:* ***Grid***
*#Question display logic:*
***If [LBC_Q8A] - Yes is selected [if LBC_Q8A == 1]***
*[Varlabel - Frequency of feelings and actions as a result of the pandemic]*

**[LMC_Q9]** How often have you done each of the following as a result of the Coronavirus (COVID-19 pandemic in the past 2 weeks? (Please select one option on each row)

| -[LMC_Q9_1] | Experienced suicidal thoughts/ feelings |
| --- | --- |
| -[LMC_Q9_2] | Deliberatley hurt myself |
| -[LMC_Q9_3] | Worried about someone close to me dying |

| <1> | Once a day or more often |
| --- | --- |
| <2> | Nearly every day |
| <3> | A few times a week |
| <6> | Passing thoughts |
| <977 fixed xor> | Don’t know |
| <933 fixed xor> | Prefer not to say |

*#option display logic:*
***[LMC_Q9_1] - If [LMC_Q8B] - Experienced suicidal thoughts/ feelings, Yes is selected***
***Or [LMC_Q9_2] - If [LMC_Q8B] - Deliberately hurt myself, Yes is selected***
***Or [LMC_Q9_3] - If [LMC_Q8B] - Worried about someone close to me dying, Yes is selected***
 ***[if LMC_Q8_1 == 1 or LMC_Q8_2 == 1 or LMC_Q8_3 == 1]***

#PAGE 13

*Base: All UK Adults 18+*
*Question type:* ***Text***

Thank you for taking part in this survey. If you've been affected by this topic and would like any more information, need advice, or support, you can go to the following place for help:


Mental Health Foundation (www.mentalhealth.org.uk)

Mind (www.Mind.org.uk)

Samaritans (www.samaritians.org)

Please click forward to complete the survey...
