# Supplementary material for: Inequalities and mental health during the Coronavirus pandemic in the UK: a mixed-methods exploration
Source: BMC Public Health. 2023 Sep 20;23:1830. doi: 10.1186/s12889-023-16523-9 (PMC10510114; doi:10.1186/s12889-023-16523-9)
Supplement: Supplementary file 2 — Additional file 2. [file 12889_2023_16523_MOESM2_ESM.docx]

**Supplementary material 2**

**Survey findings that informed FGD topic guides**

**Wave 2: *Socio-economic* *inequalities and mental health***

We conducted our survey data collection wave 2 in April 2020 (during the first UK-wide lockdown) with a total of 2221 participants. The Table S1 below shows the demographic characteristics of the participants.

Table S1. Descriptive statistics of participants at wave 2

| Participant characteristics | Category | N | Percent |
| --- | --- | --- | --- |
| Gender | Female | 1,204 | 54.21 |
|  | Male | 1,017 | 45.79 |
| Age group | 18-24 | 215 | 9.68 |
|  | 25-34 | 355 | 15.98 |
|  | 35-44 | 368 | 16.57 |
|  | 45-54 | 350 | 15.76 |
|  | 55+ | 933 | 42.01 |
| Social grade | C2DE, working | 903 | 40.66 |
|  | ABC1, middle | 1,318 | 59.34 |
| Work status | Working full time | 881 | 39.67 |
|  | Working part time | 365 | 16.43 |
|  | Full time student | 123 | 5.54 |
|  | Retired | 564 | 25.39 |
|  | Unemployed | 77 | 3.47 |
| Marital status | Not working/Other | 211 | 9.5 |
|  | Married/ Civil Partnership | 1,036 | 46.65 |
|  | Living as married | 298 | 13.42 |
|  | Separated/ Divorced | 208 | 9.37 |
|  | Widowed | 80 | 3.6 |
|  | Never Married | 584 | 26.29 |
| Country | England | 1,876 | 84.47 |
|  | Wales | 109 | 4.91 |
|  | Scotland | 172 | 7.74 |
|  | Northern Ireland | 64 | 2.88 |

**Logistic regression**

We analyse the socioeconomic inequalities during the pandemic using the survey data collected at wave 2 (corresponding to the time of Focus Group 1). Table S2 shows the logistic regression results of people’s financial concerns, worries about losing jobs, worries about having enough food to meet my/my households basic needs, worries about education or career training being interrupted, on a series of sociodemographic variables, shown in the Models 1-4 respectively. If the odds ratio is larger than 1, it means more likely to have that worry, and if the odds ratio is smaller than 1, it means less likely to have that worry. The results show that when controlling for other factors, there are no significant differences among different gender groups and people in the four devolved nations, and no obvious differences among people with different marital status. Regarding different age groups, compared with the youngest age group, older people (aged 55+) are less likely to have financial concerns, worries about losing job, and food worries; and all other age groups (aged 25+) are less likely to show concerns about education or career training being interrupted. People of middle class are less likely to have financial concerns and food worries compared with people of working class. In terms of different working status, full-time students are less likely to worry about losing jobs but are significantly showing more worries about education or career training being interrupted; retired people are less likely to have financial concerns and worries about losing job; and people who are not working are more likely to worry about having enough food to meet the basic needs for themselves or their households.

**Table S2 Socioeconomic inequalities during the pandemic (Logistic regression, odds ratio)**

|  | (1) | (2) | (3) | (4) |
| --- | --- | --- | --- | --- |
|  | Financial concerns | Worries about losing job | Food worries | Concerns about education |
| **Gender (Ref: Female)** |  |  |  |  |
| Male | 0.836 | 0.879 | 0.914 | 1.000 |
|  | (-1.71) | (-1.03) | (-0.94) | (-0.00) |
| **Age (Ref: 18-24)** |  |  |  |  |
| Age 25-34 | 0.806 | 0.630 | 0.999 | 0.363^***^ |
|  | (-0.95) | (-1.81) | (-0.00) | (-3.71) |
| Age 35-44 | 1.164 | 0.647 | 1.442 | 0.253^***^ |
|  | (0.65) | (-1.64) | (1.58) | (-4.72) |
| Age 45-54 | 0.783 | 0.583^*^ | 0.952 | 0.126^***^ |
|  | (-1.02) | (-2.00) | (-0.21) | (-6.41) |
| Age 55+ | 0.457^**^ | 0.358^***^ | 0.538^*^ | 0.039^***^ |
|  | (-3.19) | (-3.60) | (-2.57) | (-8.07) |
| **Social grade (Ref: C2DE)** |  |  |  |  |
| ABC1 | 0.564^***^ | 0.834 | 0.705^***^ | 0.864 |
|  | (-5.36) | (-1.41) | (-3.62) | (-0.90) |
| **Working status (Ref: Full-time)** |  |  |  |  |
| Working part-time | 1.102 | 0.867 | 1.187 | 1.431 |
|  | (0.66) | (-0.89) | (1.21) | (1.68) |
| Full-time student | 0.747 | 0.337^**^ | 1.006 | 7.982^***^ |
|  | (-1.07) | (-3.18) | (0.02) | (5.70) |
| Retired | 0.301^***^ | 0.045^***^ | 1.252 | 0.303 |
|  | (-6.16) | (-4.24) | (1.36) | (-1.83) |
| Unemployed | 1.368 | 0.673 | 1.418 | 1.368 |
|  | (1.11) | (-0.90) | (1.32) | (0.80) |
| Not working/Other | 0.874 | 0.653 | 1.631^**^ | 1.589 |
|  | (-0.76) | (-1.62) | (2.84) | (1.76) |
| **Marital status (Ref: Married/Civil partner)** |  |  |  |  |
| Living as married | 1.389^*^ | 1.267 | 0.861 | 1.076 |
|  | (2.14) | (1.34) | (-1.01) | (0.32) |
| Separated/Divorced | 0.899 | 0.947 | 1.036 | 1.322 |
|  | (-0.55) | (-0.21) | (0.21) | (0.86) |
| Widowed | 0.861 | 0.936 | 1.132 | 0.963 |
|  | (-0.42) | (-0.11) | (0.49) | (-0.03) |
| Never married | 1.078 | 1.274 | 0.970 | 1.488^*^ |
|  | (0.54) | (1.51) | (-0.23) | (1.98) |
| **Country (Ref: England)** |  |  |  |  |
| Wales | 0.845 | 1.136 | 1.072 | 1.302 |
|  | (-0.70) | (0.45) | (0.33) | (0.71) |
| Scotland | 1.102 | 1.161 | 0.996 | 1.120 |
|  | (0.52) | (0.67) | (-0.02) | (0.40) |
| Northern Ireland | 0.724 | 0.853 | 0.597 | 1.105 |
|  | (-1.02) | (-0.42) | (-1.76) | (0.23) |
| *N* | 1932 | 1386 | 2067 | 1248 |
| pseudo *R*^2^ | 0.107 | 0.074 | 0.029 | 0.300 |

Exponentiated coefficients; *t* statistics in parentheses

^*^ *p* < 0.05, ^**^ *p* < 0.01, ^***^ *p* < 0.001

**Wave 4: *Diverging inequalities and mental health experiences***

We conducted our survey data collection wave 4 in June 2020 (upon gradual lifting of the first UK lockdown) with a total of 4382 participants. The Table S3 below shows the demographic characteristics of the participants.

Table S3. Descriptive statistics of participants at wave 4

| Participant characteristics | Category | N | Percent |
| --- | --- | --- | --- |
| Gender | Female | 2,382 | 54.36 |
|  | Male | 2,000 | 45.64 |
| Age group | 18-24 | 338 | 7.71 |
|  | 25-34 | 671 | 15.31 |
|  | 35-44 | 742 | 16.93 |
|  | 45-54 | 780 | 17.8 |
|  | 55+ | 1,851 | 42.24 |
| Social grade | C2DE, working class | 1,707 | 38.95 |
|  | ABC1, middle class | 2,675 | 61.05 |
| Work status | Woking full time | 1,758 | 40.12 |
|  | Working part time | 585 | 13.35 |
|  | Full time student | 159 | 3.63 |
|  | Retired | 1,215 | 27.73 |
|  | Unemployed | 159 | 3.63 |
|  | Not working/Other | 506 | 11.55 |
| Marital status | Married/ Civil Partnership | 2,123 | 48.45 |
|  | Living as married | 585 | 13.35 |
|  | Separated/ Divorced | 390 | 8.9 |
|  | Widowed | 153 | 3.49 |
|  | Never Married | 1,101 | 25.13 |
| Country | England | 3,666 | 83.66 |
|  | Wales | 220 | 5.02 |
|  | Scotland | 378 | 8.63 |
|  | Northern Ireland | 118 | 2.69 |

**Logistic regression**

We analyse the diverging experiences during the pandemic using the survey data collected at wave 4 (corresponding to the time of Focus Group 2). Table S4 shows the logistic regression results of people’s financial concerns, emotions such as anxiety or worries, and loneliness, on a series of sociodemographic variables, shown in the Models 1-3 respectively. The results show that when controlling for other factors, there are no obvious differences among people in the four devolved nations. In terms of gender differences, men are less likely to feel anxious/worried and lonely, compared with women. Regarding different age groups, compared with the youngest age group, older people (aged 55+) are less likely to have financial concerns, feeling anxious or worries, or lonely; middle-aged people (aged 45-54) are also less likely to show feelings of loneliness. People of middle class are less likely to have financial concerns, but are more likely to feel anxious or worried, compared with people of working class. In terms of different working status, compared with people working full-time, people who are working part-time, unemployed and not working tend to have more financial concerns, while retired people are less likely to have financial concerns; and people who are not working are more likely to feel anxious or worried. Compared with people who are married or in a civil partnership, widowed people tend to feel less anxious or worried but are significantly more likely to feel lonely; people who are separated or divorced and those never married are also more likely to feel lonely.

**Table S4 Financial concerns and emotional experiences (Logistic regression, odds ratio)**

|  | (1) | (2) | (3) |
| --- | --- | --- | --- |
|  | Financial concerns | Anxious/worried | Lonely |
| **Gender (Ref: Female)** |  |  |  |
| Male | 0.877 | 0.473^***^ | 0.556^***^ |
|  | (-1.65) | (-11.47) | (-7.39) |
| **Age (Ref: 18-24)** |  |  |  |
| Age 25-34 | 1.179 | 1.194 | 0.865 |
|  | (0.96) | (1.10) | (-0.90) |
| Age 35-44 | 1.138 | 0.932 | 0.745 |
|  | (0.74) | (-0.44) | (-1.77) |
| Age 45-54 | 0.955 | 0.752 | 0.412^***^ |
|  | (-0.26) | (-1.74) | (-5.11) |
| Age 55+ | 0.591^**^ | 0.569^***^ | 0.365^***^ |
|  | (-2.80) | (-3.33) | (-5.45) |
| **Social grade (Ref: C2DE)** |  |  |  |
| ABC1 | 0.621^***^ | 1.153^*^ | 1.165 |
|  | (-5.86) | (2.13) | (1.90) |
| **Working status (Ref: Full-time)** |  |  |  |
| Working part-time | 1.391^**^ | 1.182 | 0.886 |
|  | (2.95) | (1.61) | (-0.99) |
| Full-time student | 0.983 | 0.820 | 0.997 |
|  | (-0.07) | (-0.98) | (-0.02) |
| Retired | 0.307^***^ | 0.900 | 1.035 |
|  | (-7.61) | (-0.98) | (0.24) |
| Unemployed | 2.929^***^ | 1.069 | 0.789 |
|  | (5.62) | (0.38) | (-1.19) |
| Not working/Other | 1.469^**^ | 1.432^**^ | 1.249 |
|  | (3.20) | (3.22) | (1.81) |
| **Marital status (Ref: Married/Civil partner)** |  |  |  |
| Living as married | 1.322^*^ | 1.086 | 1.033 |
|  | (2.44) | (0.81) | (0.25) |
| Separated/Divorced | 1.044 | 1.113 | 4.063^***^ |
|  | (0.30) | (0.93) | (11.15) |
| Widowed | 0.962 | 0.581^**^ | 4.141^***^ |
|  | (-0.13) | (-3.01) | (7.56) |
| Never married | 1.111 | 0.935 | 3.088^***^ |
|  | (1.02) | (-0.74) | (11.11) |
| **Country (Ref: England)** |  |  |  |
| Wales | 1.200 | 1.072 | 1.256 |
|  | (1.07) | (0.48) | (1.35) |
| Scotland | 0.953 | 0.957 | 1.238 |
|  | (-0.35) | (-0.40) | (1.64) |
| Northern Ireland | 0.806 | 0.657^*^ | 0.652 |
|  | (-0.91) | (-2.12) | (-1.63) |
| *N* | 3655 | 4352 | 4352 |
| pseudo *R*^2^ | 0.106 | 0.052 | 0.109 |

Exponentiated coefficients; *t* statistics in parentheses

^*^ *p* < 0.05, ^**^ *p* < 0.01, ^***^ *p* < 0.001

**Wave 6: *Resilience and coping strategies***

We had our survey data collection wave 6 in August 2020 (upon first UK lockdown fully lifted) with a total of 4584 participants. The Table S5 below shows the demographic characteristics of the participants.

Table S5. Descriptive statistics of participants at wave 6

| Participant characteristics | Category | N | Percent |
| --- | --- | --- | --- |
| Gender | Female | 2,461 | 53.69 |
|  | Male | 2,123 | 46.31 |
| Age group | 18-24 | 425 | 9.27 |
|  | 25-34 | 709 | 15.47 |
|  | 35-44 | 756 | 16.49 |
|  | 45-54 | 768 | 16.75 |
|  | 55+ | 1,926 | 42.02 |
| Social grade | C2DE, working class | 1,825 | 39.81 |
|  | ABC1, middle class | 2,759 | 60.19 |
| Work status | Woking full time | 1,719 | 37.5 |
|  | Working part time | 666 | 14.53 |
|  | Full time student | 197 | 4.3 |
|  | Retired | 1,214 | 26.48 |
|  | Unemployed | 242 | 5.28 |
|  | Not working/Other | 546 | 11.91 |
| Marital status | Married/ Civil Partnership | 2,138 | 46.64 |
|  | Living as married | 600 | 13.09 |
|  | Separated/ Divorced | 400 | 8.73 |
|  | Widowed | 191 | 4.17 |
|  | Never Married | 1,237 | 26.99 |
|  | . | 18 | 0.39 |
| Country | England | 3,830 | 83.55 |
|  | Wales | 237 | 5.17 |
|  | Scotland | 404 | 8.81 |
|  | Northern Ireland | 113 | 2.47 |

**Regression of coping**

We analyse people’s coping during the pandemic using the survey data collected at wave 6 (corresponding to the time of Focus Group 3). Table S6 shows the OLS regression results of people’s coping on a series of sociodemographic variables. The results show that when controlling for other factors, there are no significant differences among different social grades and people in the four devolved nations. In terms of different gender groups, men tend to cope better than women. Regarding different age groups, older age groups (aged 45+) tend to cope better than younger age groups. In terms of different working status, compared with people working full-time, people who are unemployed or not working tend to cope worse with the stress related to the pandemic. Compared with people who are married or in a civil partnership, people who are separated or divorced and those never married tend to cope less well.

**Table S6 Coping during the pandemic (OLS regression)**

|  | **Coping** |
| --- | --- |
| **Gender (Ref: Female)** |  |
| Male | 0.092*** |
|  | (4.02) |
| **Age (Ref: 18-24)** |  |
| Age 25-34 | 0.061 |
|  | (1.19) |
| Age 35-44 | 0.073 |
|  | (1.40) |
| Age 45-54 | 0.149*** |
|  | (2.82) |
| Age 55+ | 0.244*** |
|  | (4.38) |
| **Social grade (Ref: C2DE)** |  |
| ABC1 | 0.019 |
|  | (0.82) |
| **Working status (Ref: Full-time)** |  |
| Working part-time | -0.064* |
|  | (-1.84) |
| Full-time student | -0.009 |
|  | (-0.13) |
| Retired | 0.020 |
|  | (0.51) |
| Unemployed | -0.207*** |
|  | (-4.01) |
| Not working/Other | -0.221*** |
|  | (-5.85) |
| **Marital status (Ref: Married/Civil partner)** |  |
| Living as married | 0.012 |
|  | (0.33) |
| Separated/Divorced | -0.090** |
|  | (-2.18) |
| Widowed | -0.066 |
|  | (-1.13) |
| Never married | -0.088*** |
|  | (-2.87) |
| **Country (Ref: England)** |  |
| Wales | 0.030 |
|  | (0.59) |
| Scotland | 0.025 |
|  | (0.65) |
| Northern Ireland | -0.084 |
|  | (-1.18) |
| **Constant** | 2.862*** |
|  | (51.27) |
| **N** | 3709 |
| **R^2^** | 0.055 |
| **Adjusted R^2^** | 0.051 |
| **F** | 11.962 |

Notes. T statistics in parentheses. * p<0.10. ** p<0.05. *** p<0.01.

**Logistic regression of coping strategies**

We analyse the coping strategies during the pandemic using the survey data collected at wave 6 (corresponding to the time of Focus Group 3). Table S7 shows the logistic regression results of people’s coping strategies on a series of sociodemographic variables, shown in the Models 1-4 respectively. If the odds ratio is larger than 1, it means more likely to find that coping strategy useful, and if the odds ratio is smaller than 1, it means less likely to find that coping strategy useful. The results show that when controlling for other factors, there are no significant differences among people in the four devolved nations. Compared with women, men are less likely to express these coping strategies are useful. Regarding different age groups, compared with the youngest age group, middle-aged people (aged 45-54) are less likely to express that contacting friends are helpful ways of coping. People of middle class are more likely to express that all these coping strategies help them to cope, compared with people of working class. In terms of different working status, people who are not working are less likely to utilise these coping strategies, while retired people are more likely to find contacting family and friends are helpful ways of coping. Compared with people who are married or in a civil partnership, people who are separated or divorced, widowed, and never married are less likely to express accessing nature as useful coping strategy for them. People who are never married are also less likely to find contacting family as useful coping but more likely to think contacting friends are helpful for them to cope with stress during the pandemic.

**Table S7 Coping strategies during the pandemic (Logistic regression, odds ratio)**

|  | (1) | (2) | (3) | (4) |
| --- | --- | --- | --- | --- |
|  | Going for a walk | Visiting green space | Contacting family | Contacting friends |
| **Gender (Ref: Female)** |  |  |  |  |
| Male | 0.764^***^ | 0.789^***^ | 0.476^***^ | 0.499^***^ |
|  | (-3.82) | (-3.45) | (-10.61) | (-9.96) |
| **Age (Ref: 18-24)** |  |  |  |  |
| Age 25-34 | 0.795 | 1.072 | 1.147 | 0.988 |
|  | (-1.49) | (0.46) | (0.90) | (-0.08) |
| Age 35-44 | 0.714^*^ | 0.871 | 0.966 | 0.704^*^ |
|  | (-2.13) | (-0.90) | (-0.22) | (-2.26) |
| Age 45-54 | 0.684^*^ | 0.780 | 0.872 | 0.580^***^ |
|  | (-2.36) | (-1.57) | (-0.86) | (-3.42) |
| Age 55+ | 0.843 | 1.024 | 1.256 | 0.792 |
|  | (-1.01) | (0.14) | (1.36) | (-1.40) |
| **Social grade (Ref: C2DE)** |  |  |  |  |
| ABC1 | 1.503^***^ | 1.658^***^ | 1.288^***^ | 1.450^***^ |
|  | (5.74) | (7.23) | (3.56) | (5.22) |
| **Working status (Ref: Full-time)** |  |  |  |  |
| Working part-time | 0.901 | 1.147 | 0.960 | 1.019 |
|  | (-0.97) | (1.31) | (-0.38) | (0.18) |
| Full-time student | 0.941 | 1.242 | 1.040 | 1.360 |
|  | (-0.31) | (1.13) | (0.20) | (1.57) |
| Retired | 0.850 | 0.968 | 1.435^**^ | 1.504^***^ |
|  | (-1.33) | (-0.27) | (3.00) | (3.41) |
| Unemployed | 0.884 | 0.832 | 0.750 | 0.960 |
|  | (-0.80) | (-1.19) | (-1.81) | (-0.26) |
| Not working/Other | 0.649^***^ | 0.785^*^ | 0.907 | 0.794^*^ |
|  | (-3.81) | (-2.13) | (-0.86) | (-2.00) |
| **Marital status (Ref: Married/Civil partner)** |  |  |  |  |
| Living as married | 0.952 | 0.922 | 0.836 | 0.985 |
|  | (-0.45) | (-0.77) | (-1.67) | (-0.13) |
| Separated/Divorced | 0.698^**^ | 0.725^**^ | 1.066 | 1.162 |
|  | (-2.86) | (-2.58) | (0.50) | (1.19) |
| Widowed | 0.654^*^ | 0.673^*^ | 1.085 | 1.371 |
|  | (-2.39) | (-2.24) | (0.44) | (1.74) |
| Never married | 0.622^***^ | 0.638^***^ | 0.710^***^ | 1.238^*^ |
|  | (-5.10) | (-4.89) | (-3.69) | (2.29) |
| **Country (Ref: England)** |  |  |  |  |
| Wales | 1.265 | 0.969 | 1.234 | 1.005 |
|  | (1.49) | (-0.21) | (1.37) | (0.04) |
| Scotland | 1.124 | 1.022 | 0.958 | 1.080 |
|  | (0.97) | (0.18) | (-0.36) | (0.65) |
| Northern Ireland | 1.404 | 1.143 | 1.089 | 1.363 |
|  | (1.50) | (0.62) | (0.39) | (1.43) |
| *N* | 3843 | 3843 | 3843 | 3843 |
| pseudo *R*^2^ | 0.024 | 0.027 | 0.049 | 0.044 |

Exponentiated coefficients; *t* statistics in parentheses

^*^ *p* < 0.05, ^**^ *p* < 0.01, ^***^ *p* < 0.001
