# Supplementary material for: Inequalities and mental health during the Coronavirus pandemic in the UK: a mixed-methods exploration
Source: BMC Public Health. 2023 Sep 20;23:1830. doi: 10.1186/s12889-023-16523-9 (PMC10510114; doi:10.1186/s12889-023-16523-9)
Supplement: Supplementary file 3 — Additional file 3. [file 12889_2023_16523_MOESM3_ESM.docx]

**~ CORONAVIRUS: MENTAL HEALTH IN THE PANDEMIC STUDY ~**

**FOCUS GROUP DISCUSSION TOPIC GUIDES:**

**INEQUALITIES AND MENTAL HEALTH DURING THE PANDEMIC**

**Focus Group Discussion 1: Financial inequalities and mental health**

| **SECTION 1: BACKGROUND & INTRODUCTIONS** |  |  |
| --- | --- | --- |
| **1. Background - Facilitators**     - Introduce self - Roles of facilitators within the group discussion |  |  |
| **2. Housekeeping & Introductions**   - Housekeeping (*The FGD will be recorded - then transcribed - to look at your responses in detail, but everything you say will be confidential and your name will not be identified in any findings)*   - Time of comfort break   - Chat function   - Attention indicator      - The main objective of the FGD discussion today:   *“****To gain a deeper understanding of the current Coronavirus pandemic and the impact that financial inequalities might have had on mental health”***   - Introductions - *Explain structure* |  |  |
| **SECTION 2: DIVERGENCE OF EXPEREINCE** |  |  |
| - What groups do you think are most impacted by financial inequalities - Presentation of Survey findings linked to Financial Inequalities   COMFORT BREACK   - Reflections/reactions after the presentation, any surprises? - Where would you like to see changes (in terms of policy, government 's recovery plan etc)? - How can these be achieved   SECTION 3: CLOSING |  |  |
| - Wrap up of session - Is there anything else you want to say, or anything you feel we have not discussed today that you’d like to add |  |  |
| - - Brief chat around what happens next (informing briefing; press release etc ) |  |  |
|  |  |  |

**Focus Group Discussion 2: Divergence of mental health experiences**

| **SECTION 1: BACKGROUND & INTRODUCTIONS** |  |  |
| --- | --- | --- |
| **1. Background - Facilitators**     - Introduce self - Roles of facilitators within the group discussion |  |  |
| **2. Housekeeping & Introductions**   - Housekeeping (*The FGD will be recorded - then transcribed - to look at your responses in detail, but everything you say will be confidential and your name will not be identified in any findings)*   - Time of comfort break   - Chat function   - Attention indicator      - The main objective of the FGD discussion today:   *“****To gain a deeper understanding of the current Coronavirus pandemic and related measures in the UK, how these impact people’s mental wellbeing, who are most impacted, and how/why experiences may differ, and what can be done’***   - Introductions - *Explain structure* |  |  |
| **SECTION 2: DIVERGENCE OF EXPERIENCE** |  |  |
| - What groups do you think are most impacted by divergence of experience - Presentation of Survey findings linked to Experience of the pandemic in different groups   COMFORT BREACK   - Reflections/reactions after the presentation, any surprises? - Where would you like to see changes (in terms of policy, government 's recovery plan etc)? - How can these be achieved   SECTION 3: CLOSING |  |  |
| - Wrap up of session - Is there anything else you want to say, or anything you feel we have not discussed today that you’d like to add - Brief chat around what happens next (informing briefing; press release etc ) |  |  |

| **Focus Group Discussion 3: Coping strategies**   \| **SECTION 1: BACKGROUND & INTRODUCTIONS** \|  \|  \| \| --- \| --- \| --- \| \| **1. Background - Facilitator**     - Introduce self - Roles of facilitators within the group discussion \|  \|  \| \| **2. Housekeeping & Introductions**   - Housekeeping (*The FGD will be recorded - then transcribed - to look at your responses in detail, but everything you say will be confidential and your name will not be identified in any findings)*   - Time of comfort break   - Chat function   - Attention indicator      - The main objective of the FGD discussion today:   *“****To gain a deeper understanding of what has helped people deal with the stress of lockdown and lifting of restrictions….why some people dealt with everything better than others….and what have we learned about ourselves and our community”***   - Respondent Introductions (given new people) - *Explain structure: First half of the group we’re going to chat around your own experiences during lockdown and then in the second half we’re going to explore in more detail how different groups may have had different experiences* \|  \|  \| \| **SECTION 2: GENERAL LEVEL OF RESILIENCE & COPING WITHIN GROUP** \|  \|  \| \| - Past 5 months timeline – from complete lockdown to re-opening of economy and schools   - What **one word or phrase** would you use to describe how you feel you have dealt with your own experiences during the last 5 months   - Write this down in big letters on your pad and then hold it up to the screen or just go round the room     - Why pick this phrase / word?     - (Open discussion around reasons why / specific lockdown issues relating to own circumstances to gather context for rest of session) \|  \|  \| \| **SECTION 3: STATEMENTS RELATING TO RESILIENCE AND COPING STRATEGIES** \|  \|  \| \| *(All statements Link back to briefing and the positive ways of coping with stress through the Five Ways to Wellbeing developed by the New Economics Foundation)*  **Statement 1: (***Slide on screen )*  ***HAVING DAILY CONTACT WITH OTHERS HELPED ME DEAL WITH THE STRESS RELATED TO THE CORONAVIRUS PANDEMIC*** \|  \|  \| \| **Statement 2: (***Slide on screen )*  ***GETTING DAILY EXERCISE / DOING SOME SORT OF PHYSICAL ACTIVITY / BEING IN GREEN SPACE HELPED MY DEAL WITH THE STRESS RELATED TO THE CORONAVIRUS PANDEMIC*** \|  \|  \| \| **Statement 3: (***Slide on screen)*  ***LIMITING MY EXPOSURE TO THE NEWS / SOCIAL MEDIA AROUND CORONAVIRUS HELPED ME MANAGE MY OWN ANXIETY*** \|  \|  \| \| **Statement 4: (***Slide on screen)*  ***ENGAGING IN A HOBBY / DEVELOPED A NEW SKILL DURING LOCKDOWN WAS IMPORTANT TO ME*** \|  \|  \| \| **Statement 5: (***Slide on screen)*  ***VOLUNTEERING / HELPING MY LOCAL COMMUNITY ASSISTED ME DURING LOCKDOWN*** \|  \|  \| \| **COMFORT BREAK** \|  \|  \| \| **SECTION 4: NEGATIVE COPING STRATEGIES**   - We have been talking about all the positive ways that people could use to help them cope with the stress of the pandemic. However, what about those less positive ways:   - What ways of dealing with stress of the pandemic would you consider not helpful? (probe here on alcohol / over-eating / smoking / drugs)   - Why do some people find these more helpful with dealing with the stress than others? What factors impact on this do you think? \|  \|  \| \| **SECTION 6: RETROSPECTIVE**   - Thinking back now to lockdown – in retrospect - what one thing would you have done differently to make the experience different for you?   Is there anything that any of you have been doing to cope that you will continue to do ? \|  \|  \| \| **SECTION 7: COMING OUT OF LOCKDOWN: SUPPORTING THEIR OWN MENTAL HEALTH & WELLBEING**   - Now that restrictions are being lifted what are you most looking forward to? Been enjoying the most? Why? - Is there anything about the restrictions being lifted that make you anxious / that may affect your mental health. Why? - What would help you continue to manage your stress around the pandemic?   - What community support would help?   - How can the Government help manage people cope better – what do they need to put in place now? \|  \|  \| \| **SECTION 8: MOVING FORWARD**   - How would you like things to change / improved / resolved going forward over the next 6months – 1 year? What support do you (or your family) need to support your OWN mental health & wellbeing? What would be the ideal solution? Probe on the following areas   - Health service needs   - Practical help   - Social support (including childcare)   - Psychological support \|  \|  \| \| **Closing**   - Facilitator   - Survey findings linked to resilience and coping… (**SS to provide some data on A4 and some charts)**   - Brief chat around what happens next (informing briefing; press 8^th^ October; WMHD etc) - Is there anything else you want to say, or anything you feel we have not discussed today that you’d like to add? \|  \|  \| |  |  |
| --- | --- | --- | --- | --- | --- | --- | --- | --- | --- | --- | --- | --- | --- | --- | --- | --- | --- | --- | --- | --- | --- | --- | --- | --- | --- | --- | --- | --- | --- | --- | --- | --- | --- | --- | --- | --- | --- | --- | --- | --- | --- | --- | --- | --- | --- | --- | --- | --- | --- | --- | --- | --- | --- |
|  |  |  |
